# Building a dementia care effectiveness trial to facilitate and anticipate widespread intervention adoption

**DOI:** 10.1002/alz.092491

**Published:** 2025-01-09

**Authors:** Richard H Fortinsky, Julie T Robison

**Affiliations:** ^1^ University of Connecticut School of Medicine, Farmington, CT USA

## Abstract

**Background:**

This presentation maps the journey of an evidence‐based dementia care program from effectiveness trial design to adoption by policymakers as an ongoing program available to eligible older adults living at home with dementia and their care partners. Care of People with Dementia in their Environments (COPE) is a non‐pharmacologic program that seeks to maximize physical function and quality of life in people with dementia and build dementia management skills in care partners. Efficacy trial results showed that, compared to controls, people with dementia receiving COPE had greater functional independence in activities of daily living and care partners receiving COPE reported improved well‐being and increased confidence using dementia management skills.

**Methods:**

From 2014‐2020, COPE was tested in Connecticut, USA, using an effectiveness‐implementation hybrid design. The target population was people with dementia receiving services from Connecticut’s Medicaid‐funded home and community‐based services (HCBS) program and their care partners. Several study features were designed to anticipate the possibility that COPE might be adopted for widespread use if the study were successful. First, a translational advisory committee met annually to help guide the study and interpret findings with an eye toward offering COPE within Connecticut’s HCBS program throughout the state. Advisory committee members included leaders from Connecticut’s Medicaid program and from the state’s largest HCBS service coordination organization. Second, this service coordination organization co‐designed study recruitment procedures with the study team, including use of its electronic health record system to identify and invite people with dementia and care partners to join the study. Third, qualitative data were used to give voice to stakeholders about how well COPE was implemented within the HCBS program workflow. Fourth, extensive cost data were captured enabling economic analyses to be presented to policymakers.

**Findings:**

Care partners receiving COPE reported more improved well‐being, and total healthcare costs were lower among older adults receiving COPE, compared to those not receiving COPE.

**Conclusions:**

Based on study findings, COPE is being incorporated into Connecticut’s HCBS program pursuant to public policy opportunities that arose in the wake of the COVID pandemic, which has provided funding for a variety of new Medicaid HCBS initiatives.

